# High Efficient Expression, Purification, and Functional Characterization of Native Human Epidermal Growth Factor in* Escherichia coli*

**DOI:** 10.1155/2016/3758941

**Published:** 2016-09-28

**Authors:** Yi Ma, Jieying Yu, Jinglian Lin, Shaomin Wu, Shan Li, Jufang Wang

**Affiliations:** ^1^School of Bioscience and Bioengineering, South China University of Technology, Guangzhou 510006, China; ^2^Guangdong Province Key Laboratory of Fermentation and Enzyme Engineering, South China University of Technology, Guangzhou 510006, China

## Abstract

Human epidermal growth factor (hEGF) is a small, mitotic growth polypeptide that promotes the proliferation of various cells and is widely applied in clinical practices. However, high efficient expression of native hEGF in* Escherichia coli* has not been successful, since three disulfide bonds in monomer hEGF made it unable to fold into correct 3D structure using* in vivo* system. To tackle this problem, we fused* Mxe* GyrA intein (Mxe) at the C-terminal of hEGF followed by small ubiquitin-related modifier (SUMO) and 10x His-tag to construct a chimeric protein hEGF-Mxe-SUMO-H_10_. The fusion protein was highly expressed at the concentration of 281 mg/L and up to 59.5% of the total cellular soluble proteins. The fusion protein was purified by affinity chromatography and 29.4 mg/L of native hEGF can be released by thiol induced N-terminal cleavage without any proteases. The mitotic activity in Balb/c 3T3 cells is proliferated by commercial and recombinant hEGF measured with methylthiazolyldiphenyl-tetrazolium bromide (MTT) assay which indicated that recombinant hEGF protein stimulates the cell proliferation similar to commercial protein. This study significantly improved the yield and reduced the cost of hEGF in the recombinant* E. coli* system and could be a better strategy to produce native hEGF for pharmaceutical development.

## 1. Introduction

hEGF, a polypeptide hormone in human body, is widely used in medicine and cosmetics industry [1]. The polypeptide not only stimulates cell proliferation, differentiation, and migration, but also plays an extremely important role in wound healing, organ generation, and cell signal transduction [2].* hEGF* gene has been successfully expressed in various heterologous expression systems.* E. coli*, one of the preferred organisms for heterologous protein expression, is regarded as the simplest and cheapest system to produce the commercial recombinant hEGF. However, with three intramolecular disulfide bonds, native hEGF (without additional amino acid residues) is impossible to fold correctly and be expressed solubly in prokaryotic expression system. Various tag protein fusion systems, for instance, 6x Histidine, thioredoxin, and glutathione-S-transferase, have been used to express and purify recombinant hEGF; however these procedures were not efficient in soluble expression, tag cleavage, and purification [3]. Extracellular expression of bioactive hEGF was studied in* E. coli* with the aid of the signal peptides [4, 5] and in several eukaryotic systems such as* Saccharomyces cerevisiae* [6],* Yarrowia lipolytica* [7], and* Hansenula polymorpha* [8], which significantly decreased the yield of hEGF compared to that with intracellular expression strategy. With intense interest to hEGF, studies on biosynthesis of hEGF have never been stopped. In order to solve the above-mentioned problems, especially inefficient soluble expression, Su et al. adopted an effective SUMO fusion strategy [9] which has been widely applied for soluble expression of target proteins to produce and purify hEGF fused with His-tag [10]. However, SUMO and affinity tag based expression system require the SUMO protease to cleave the SUMO tag in the purification of interested proteins [11]. This protease addition not only is costly, but also requires an extra step to separate protease from the purified hEGF protein. In this paper, native hEGF was expressed as a soluble form in* E. coli* by protein fusion strategy. The activity of recombinant hEGF was identified by the MTT assay.

The inteins are internal protein-splicing elements that self-excise themselves from precursor proteins and catalyze ligation of the flanking peptides together [12]. Based on this special mechanism, many intein-mediated applications were developed in protein refolding [13], purification [14], and ligation [15]. Zhang et al. developed* Ssp* dnaB intein fusion system to produce and purify hEGF, but the hEGF expressed as inclusion bodies and uncontrolled cleavage induced by pH presents major problems [16]. To overcome those problems, a novel purification procedure using* Mxe* GyrA intein instead of* Ssp* dnaB intein has been set up for preventing self-cleavage* in vivo* [17]. The* Mxe* GyrA intein system consisting of 198 amino acids was first reported as in-frame insertion for protein splicing nearly 20 years ago [18]. This self-chimeric system is capable of producing a native target protein with an unmodified C-terminal thioester and facilitating the expression and purification of interested proteins [19, 20].

In this study,* hEGF* gene was used in conjunction with intein, SUMO, and Histidine affinity tag to achieve soluble expression in* E. coli* followed by efficient purification and activity assay. The recombinant plasmid hEGF-Mxe-SUMO-His_10_ was transformed into BL21(DE3). About 29.4 mg/L of bioactive hEGF was obtained after C-terminal intein cleavage induced by 20 mM DTT. This novel method significantly improved the expression level of hEGF in the soluble fraction in* E. coli in vivo* system, making it easier to be purified and produce native and bioactive recombinant hEGF containing multiple intramolecular disulfide bonds in reduction system.

## 2. Materials and Methods

### 2.1. Strains, Plasmids, Enzymes, and Reagents

Competent* E. coli* cells* DH5α* and BL21(DE3) were purchased from TIANGEN Biotech (Beijing, China). Plasmids pTWIN1, pET28a-SUMO, and pET21a were preserved by our own laboratory. Restriction endonucleases* Nde* I and* Xho* I were purchased from Thermo Fisher Scientific Co., Ltd. (Shanghai, China). Prime STAR HS (Premix) LA Taq, DNA ladder Marker, and kits for DNA manipulation were purchased from TAKARA Biotechnology Co., Ltd. (Dalian, China). Protein ladder marker was obtained from Thermo Fisher Scientific (CA, USA).* Pfu* DNA polymerase was purchased from Promega (Madison, USA).* hEGF* coding region was synthesized by Huada Genomics Institute Co., Ltd. (Shenzhen, China). Commercial recombinant hEGF was purchased from Peprotech Co., Ltd. (Rocky Hill, USA). Mouse fibroblast Balb/c 3T3 cells were kindly given by Professor Yadong Huang from Jinan University. All chemicals used in this study were of analytical grade.

### 2.2. Construction of Expression Vector

The 165 bp* hEGF* coding region was chemically synthesized. The 594 bp* Mxe* GyrA gene and 297 bp* SUMO* gene were cloned from plasmid pTWIN1 and pET28a-SUMO, respectively. The coding regions using Prime STAR HS DNA Polymerase were amplified with the following primers: the forward primer used for* hEGF* was 5′-GGAATTC**CATATG**AATAGTGACTCTGAATGTCC-3′; the reverse primer for* hEGF* was 5′-CC**CTCGAG**AGGCGCAGTTCCCACCACTT-3′. Restriction sites* Nde* I and* Xho* I used for subsequent amplification are shown in bold and underline. The restricted PCR products were inserted into modified vector pET21a yielding the plasmid pET-hEGF with a C-terminal poly(His)_10_ tag. For the restriction free cloning, the standard strategy was performed to construct fusion gene in pET-hEGF-SUMO-H_10_ using the forward primer: 5′-TGAAGTGGTGGGAACTGCGCTCGGACTCAGAAGTCAATCA-3′ and the reverse primer: 5′-TGGTGGTGGTGGTGCTCGAGACCTCCAATCTGTTCGCGGT-3′, but in the final expression vector pET-hEGF-Mxe-SUMO-H_10_ using the forward primer: 5′-CTGAAGTGGTGGGAACTGCGCTGCATCACGGGAGATGCACTA-3′ and the reverse primer: 5′-CTTGATTGACTTCTGAGTCCGAAGCGTGGCTGACGAACCCGTT-3′. The positive clones were identified by colony PCR and confirmed by DNA sequence analysis.

### 2.3. Expression and Analysis of Recombinant Fusion Protein hEGF-Mxe-SUMO-H_10_ in Shake Flasks

Plasmid pET-hEGF-Mxe-SUMO-H_10_ was transformed into* E. coli* BL21(DE3) (Novagen, Madison, WI, USA). The recombinant bacteria were induced to express recombinant hEGF-Mxe-SUMO-H_10_ by adding isopropyl-*β*-D-thiogalactopyranoside (IPTG) at a final concentration of 1 mM to a culture with OD_600_ of approximately 0.6–0.8 and incubating at 37°C for 4 h. Cell culture (1 L) was separated by centrifugation at 5000 ×g for 20 min. The cell pellets were resuspended in 35 mL of binding buffer (20 mM Tris-HCl, 500 mM NaCl, 20% Glycerin, 20 mM imidazole, pH 8.0) and lysed with 450 sonication pulses (400 W, 3 s with a 5 s interval) cooled in ice water bath. The suspension was centrifuged (11,000 ×g at 4°C for 30 min) and passed through a 0.22 *μ*m filter following by applied to a 1 mL HiTrap™ Chelating HP column (GE Healthcare, Piscataway, NJ, USA). The fusion protein hEGF-Mxe-SUMO-H_10_ was purified using the standard nickel affinity chromatography procedure and washed with 5 column volumes each of 50 mM, 100 mM, 150 mM, and 200 mM imidazole in column buffer (20 mM Tris-HCl, 500 mM NaCl, 20% glycerol, pH 8.0). The target protein was then eluted with column buffer supplemented with 500 mM imidazole using an ÄKTA purifier system (Amersham Pharmacia Biotech, Sweden) at flow rates of 5 mL/min. The purity of the proteins was evaluated using SDS-PAGE and Western blot and the concentration was determined by a BCA Protein Assay Kit (Sangon, Shanghai, China). For SDS gel analysis, protein samples were dissolved in SDS sample buffer and loaded on 15% (w/v) Tris glycine SDS-PAGE gels and stained with Coomassie Blue. For Western blot analysis, the proteins were transferred to a 0.45 m polyvinylidene difluoride (PVDF) membrane (Millipore) by wet Western blot for 30 min at 100 V. Membranes were blocked for 2 h at room temperature (RT) in the blocking buffer containing TBST buffer (150 mM NaCl, 10 mM Tris-HCl in H_2_O, pH 7.6, and 0.1% Tween 20) and 5% skim milk powder (Fluka, Sigma). The membrane was blocked in 50 mL of TBST buffer with 2.5 g of milk powder overnight at 4°C, washed three times at RT with 25 mL of TBST buffer, and incubated with the primary antibody to penta-His (Qiagen, Germany) at 1 : 2000 dilution at 4°C. The membrane was washed three times with 25 mL of TBST buffer at RT. The membrane was further incubated with the secondary antibody goat anti-mouse IgG horseradish peroxidase conjugate (Sigma, St. Louis, MI, USA) at a 1 : 5000 dilution in TBST buffer for 1.5 h at RT. The membrane was washed three times subsequently with TBST buffer at RT. Finally, the blots were analyzed by Chemiluminescence Imaging System ChemiScope 3600 (CliNX Science Instruments, Shanghai, China).

### 2.4. Cleavage of hEGF-Mxe-SUMO-H_10_ and Purification of Native hEGF

The purified hEGF-Mxe-SUMO-H_10_ was dialyzed in Binding Buffer (0.003 M KCl, 1.5 mM KH_2_PO_4_, 8 mM Na_2_HPO_4_, 500 mM NaCl, and 20 mM imidazole, pH 7.4) overnight at 4°C and transferred to Cleavage Buffer (0.003 M KCl, 1.5 mM KH_2_PO_4_, 8 mM Na_2_HPO_4_, 500 mM NaCl, and 20 mM imidazole and 20 mM dithiothreitol (DTT), pH 7.4) by ultrafiltration. After incubation at RT for 12 h, protein hEGF-Mxe-SUMO-H_10_ was dialyzed in Binding Buffer with 1 kD MWCO Millipore membrane (Bedford, MA, USA) generating native hEGF and Mxe-SUMO-H_10_. The cleaved sample was loaded on Ni-NTA resin to obtain native hEGF which was further concentrated employing ultrafilter with 1 kD MWCO membrane at 4°C. The immunogenic activity of recombinant native hEGF was confirmed by Western blot as described above. The concentration of recombinant native hEGF was calculated by Bradford method. The Mxe-SUMO-H_10_ protein bound to the Ni-NTA resin was eluted as described above.

### 2.5. Native hEGF Biological Activity Assay

MTT assay was performed to test the bioactivity of hEGF promoting proliferation of Balb/c 3T3 cell grown on medium 1640 supplemented with 100 *μ*g/mL streptomycin, 100 U/mL ampicillin, and 10% fetal bovine serum. Balb/c 3T3 cell at a density of 1 × 10^5^ cells/mL was seeded in basal media on sterile 96-well tissue culture plate (Corning, NY, USA) incubated with 100 *μ*L/well at 37°C and 5% CO_2_ for 36 h. When the culture achieved the mid-logarithm phase, cells were transferred to new 96-well plate (5 × 10^4^ cells/mL) and incubated for 24 h in medium 1640 containing the above supplements following by replacing medium 1640 with 0.4% fetal bovine serum and the cells were incubated for 24 h. Balb/c 3T3 cells were supplemented with recombinant native hEGF or commercial hEGF with different concentrations (from 0.39 to 25 *μ*g/mL) and incubated for 64~72 h. After 20 *μ*L/well MTT solutions were added to cells, the plates were incubated for additional 5 h at 37°C and 5% CO_2_. After discarding the medium, 100 *μ*L dimethyl sulfoxide was added to each well. The plate was kept at RT for 20 min. The absorbance was measured immediately at a wavelength of 570 nm using an Infinite® M200 pro microplate reader (Tecan, Männedorf, Switzerland). The curve of absorbance values on *y*-axis and the concentrations of growth factor on *x*-axis were plotted.

## 3. Results

### 3.1. Cloning of hEGF-H_10_, hEGF-SUMO-H_10_, and hEGF-Mxe-SUMO-H_10_

**Figure 1:**
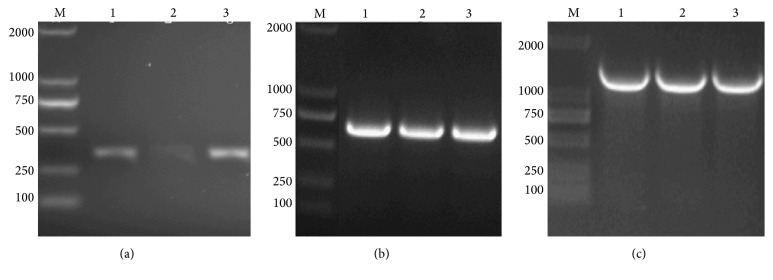
Identification of recombinant plasmids by colony PCR. M: 2 kb ladder marker; (a) lanes 1–3:* hEGF-H*_*10*_ fragment; (b) lanes 1–3:* hEGF-SUMO-H*_*10*_ fragment; (c) lanes 1–3:* hEGF-Mxe-SUMO-H*_*10*_ fragment.

Bands corresponding to* hEGF-H*_*10*_,* hEGF-SUMO-H*_*10*_, and* hEGF-Mxe-SUMO-H*_*10*_ were detected on 1.5% (w/v) agarose gel by colony PCR of recombinant plasmids (Figure 1), demonstrating that* hEGF-H*_*10*_,* hEGF-SUMO-H*_*10*_, and* hEGF-Mxe-SUMO-H*_*10*_ genes were successfully inserted into pET21a vectors, respectively.

### 3.2. Expression of Recombinant Proteins hEGF-SUMO-H_10_ and hEGF-Mxe-SUMO-H_10_

**Figure 2:**
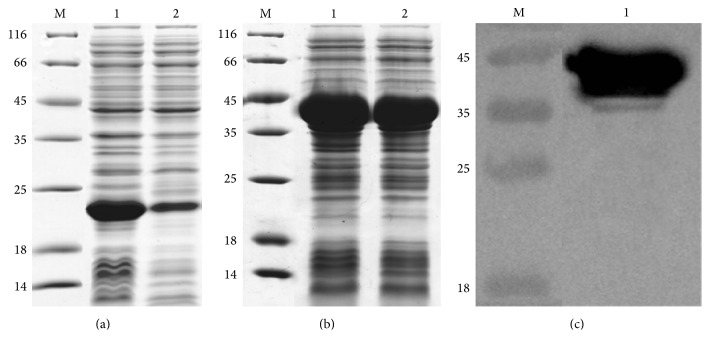
Expression of recombinant hEGF-SUMO-H_10_ and hEGF-Mxe-SUMO-H_10_. M: protein ladder marker shown in kDa on the left sides of panels, (a) SDS-PAGE analysis of the recombinant hEGF-SUMO-H_10_ produced in BL21(DE3), lane 1: whole cell sample after induction for 4 h, lane 2: supernatant sample after induction for 4 h; (b) SDS-PAGE analysis of the recombinant hEGF-Mxe-SUMO-H_10_ produced in BL21(DE3), lane 1: whole cell sample after induction for 4 h, lane 2: supernatant sample after induction for 4 h; (c) Western blot analysis of recombinant hEGF-Mxe-SUMO-H_10_ produced in BL21(DE3), lane 1: supernatant sample after induction for 4 h.

After OD_600_ of culture reached mid-logarithm time,* E. coli* cells containing recombinant proteins SUMO-hEGF-H_10_ or hEGF-Mxe-SUMO-H_10_ were induced by the addition of 0.6 mmol/L IPTG. The hEGF-SUMO-H_10_ or hEGF-Mxe-SUMO-H_10_ was expressed as C-terminal 10x His-tag fusion proteins in BL21(DE3). Both proteins could be detected by Coomassie Blue staining as a prominent band with an apparent molecular mass of 19 kDa for hEGF-SUMO-H_10_ and 40 kDa for hEGF-Mxe-SUMO-H_10_ after separation by SDS-PAGE (Figure 2). The yields were estimated for hEGF-SUMO-H_10_ to ~136 mg/L but for the part of hEGF-SUMO-H_10_ in hEGF-Mxe-SUMO-H_10_ to ~281 mg/L which indicated that* Mxe* GryA intein could obviously increase the expression level of the fusion hEGF-SUMO-H_10_ protein in* E. coli* (Figure 2). The soluble fraction of hEGF-SUMO-H_10_ in whole cell lysate was 19.4% at 37°C, while that of hEGF-Mxe-SUMO-H_10_ was 75.6% at the same temperature which demonstrated that* Mxe* GryA intein could effectively facilitate the soluble expression of the fusion protein hEGF-Mxe-SUMO-H_10_ in* E. coli* (Figure 2).

### 3.3. Purification of Recombinant Protein hEGF-Mxe-SUMO-H_10_

**Figure 3:**
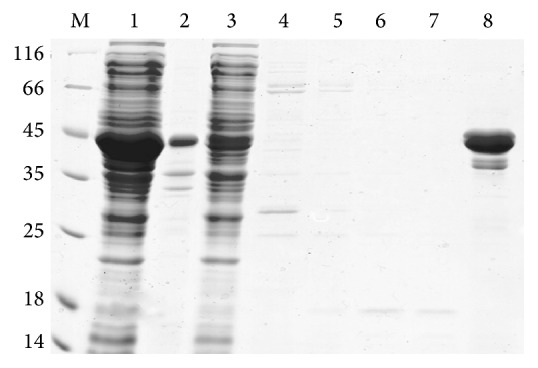
SDS-PAGE analysis of recombinant hEGF-Mxe-SUMO-H_10_ purified by one-step affinity chromatography. M: protein ladder marker shown in kDa on the left side of the panel; lane 1: supernatant sample after induction for 4 h; lane 2: precipitate sample after induction for 4 h; lane 3: flow through solution; lanes 4–8: five different elution buffer with 50 mM, 100 mM, 150 mM, 200 mM, and 500 mM imidazole buffer, respectively.

The fusion protein hEGF-Mxe-SUMO-H_10_ containing C-terminal poly(His)_10_ purification tag in the supernatant fraction of cell lysate by ultrasonic disruption was loaded to Ni-NTA column purified by affinity chromatography. Apparent pure protein sample was obtained after one step affinity purification (Figure 3). The final yield of the purified proteins was ~281 mg/L and the total purity of hEGF-Mxe-SUMO-H_10_ prepared using this method was >90% (Figure 3).

### 3.4. Cleavage of Recombinant Protein hEGF-Mxe-SUMO-H_10_ and Purification of Native hEGF

**Figure 4:**
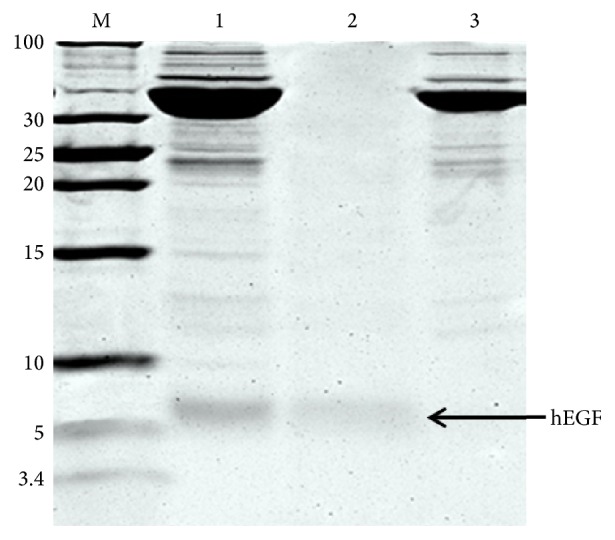
Tricine-SDS-PAGE analysis of purified recombinant hEGF prepared by self-cleavage treated with thiol DTT and one-step affinity chromatography. M: protein ladder marker shown in kDa on the left side of the panel; lane 1: supernatant solution after treatment in 20 mM DTT for 12 h; lane 2: the purified hEGF in flow through solution of the Ni-NTA column; lane 3: eluted solution with 500 mM imidazole.

Fusion protein hEGF-Mxe-SUMO-H_10_ was successfully expressed in* E. coli* BL21(DE3). The* Mxe* GyrA intein in the purified fusion protein was hydrolyzed by thiol-induced cleavage to generate the product native hEGF from recombinant protein hEGF-Mxe-SUMO-H_10_. Efficient splicing was observed after induction with 20 mM DTT at RT for 4 h and the Mxe-SUMO-H_10_ protein with a C-terminal poly(His)_10_ tag was binding to Ni-NTA resin. The native hEGF corresponding a clear band to 6 kDa in the flow through fractions was observed by Tricine-SDS-PAGE analysis and indicated with black arrow (Figure 4). The final yield of native hEGF is ~29.4 mg/L and the purified protein could be preserved without remarkable loss of activity at 80°C for months.

### 3.5. Mitotic Activity of Native hEGF

**Figure 5:**
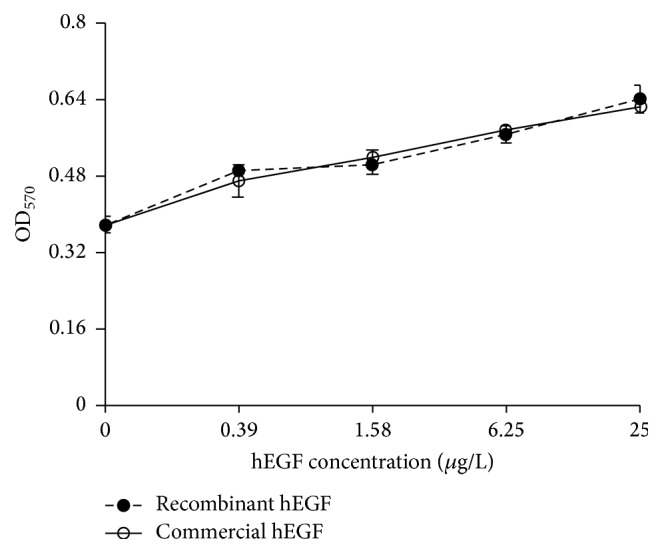
The stimulation effect of recombinant hEGF and commercial hEGF on Balb/c 3T3 cells.

The mitotic activity of native hEGF cleaved from hEGF-Mxe-SUMO-H_10_ fusion protein was in contrast to that of the commercial hEGF (Figure 5). Biological activity of the hEGF proteins was detected to assess its effectiveness in promoting Balb/c 3T3 cell proliferation. MTT assay has shown that cell proliferation enhanced after treatment with different concentrations of hEGF. The rate of cell proliferation is 1.32, 1.3, 1.43, and 1.62 times higher than that of the negative control from 0.39 to 25 *μ*g/mL of native hEGF, respectively, with *P* value < 0.05. Our result of mitotic assay indicated that hEGF protein produced by this procedure stimulates the cell proliferation similar to the commercial protein.

## 4. Discussion

hEGF is an effective stimulator to promote proliferation of a wide range of cell types, such as endothelial cell, epithelial cell, and fibroblast cell resulting in a prospective cut healing agent for various corneal and skin wounds [21]. Therefore, the market demand of hEGF has become huge and booming in recent years.* E. coli* is considered to be the preferred host for industrial production of recombinant proteins, since it has many advantages among various expression platforms, for example, robust growth rate, low cost, technical simplicity, ease of scale-up, and high capacity for heterologous protein expression [22]. However, hEGF with three disulfide bridges cannot be produced as soluble, active, and correctly folded protein in intracellular environment of* E. coli*, even fused with various protein partners which have been developed to promote the production of properly folded recombinant hEGF [3]. Since SUMO acts as a solubility enhancer, it has been frequently employed as an effective fusion partner for preventing degradation and promoting refolding of recombinant proteins [10]. SUMO can promote the translocation of partner proteins from the cytosol to the nucleus, thereby reducing the concentration of the target proteins in the protease-rich cytosol to protect against proteolytic degradation [23]. Due to the highly hydrophilic surface and hydrophobic core, SUMO acts as a nucleation site to enhance the solubility of the target protein and exert detergent-like effect on insoluble proteins [24]. Several valuable and hard-to-express proteins have been expressed successfully in* E. coli* using SUMO fusion system [25]. However, this system requires proteolytic cleavage to remove the tag, which leads problems encountered with low yield, precipitation of the target protein, expense of proteases, labor-cost optimization of cleavage and proteases removal conditions, and failure to recover active, intact protein [26]. Another main problem associated with the cleavage of fusion proteins is the production of nonnative target proteins which contain an N-terminal proline [27].

The intein fusion system with the inducible self-cleavage activity was used to generate free target protein which was expressed as insoluble inclusion bodies; thus it often requires protein solubilization and refolding to obtain active protein [28]. The widely used pTWIN vector contains two engineered mini-inteins:* Ssp* DnaB intein undergoes C-terminal cleavage while* Mxe* GyrA intein allows N-terminal cleavage [29]. The disadvantages to* Ssp* DnaB intein are the low cleavage efficiency and specificity which are influenced by the second and third amino acid residues at the N-terminus of the target protein and the deviations of pH value in host intracellular environment [30]. In the presence of thiol nucleophile, for instance, *β*-mercaptoethanol, cysteine, or DTT, the intein* Mxe* GyrA fused directly to the N-terminus of the target protein conducts specific self-cleavage resulting in producing the target protein without any extra nonnative residues [31]. The reasons for* Mxe* GyrA intein fusion system to enhance expression of hEGF are not known since it is the first time to find this system could increase the yield of target protein. We speculated that* Mxe* GyrA intein is highly stable and resistant to heat and proteolysis* in vivo* system as the* Mxe* GyrA intein is complete absence of endonuclease domain [32]. Attachment of a highly stable structure in fusion protein helps to stabilize and increase the production of recombinant protein. In addition, the inner core of* Mxe* GyrA intein is unusual hydrophobic while the outer surface is comparatively hydrophilic. This hypothesis may explain why* Mxe* GyrA intein helps to increase yield of recombinant proteins in* E. coli*.

In the present study, we successfully extended C-terminus of hEGF with intein* Mxe* GryA and His-tagged SUMO, leading to a significant increase in solubility and expression level of hEGF protein. Proteins without His-tag in cell lysate were removed from Ni-NTA affinity column. Since the Mxe-SUMO-H_10_ fusion protein bears a C-terminal poly(His)_10_ tag, the cleaved mixed proteins could be reloaded to the Ni-NTA resin to obtain purified native hEGF from cleaved Mxe-SUMO-H_10_ fusion protein and the uncleaved fusion protein, whose purity was higher than 97%. The outcomes of the expression and mitotic activity demonstrated that the C-terminal SUMO fused with* Mxe* GyrA intein could significantly improve the expression level and efficiently facilitate the correct folding of hEGF. The final yield of recombinant native hEGF is 29.4 mg/L, which is much higher than previous published strategies. For instance, Oka et al. produced 2.4 mg/L hEGF secreted from* E. coli* [4]. The yields of fusion hEGF expressed in intracellular environment of* E. coli* are less than 17 mg/L [3, 9]. Heo et al. showed a yield of 0.57 mg/L from Eukaryotic expression system* Hansenula polymorpha* [8]. Therefore, this new approach may be applied to industrial-scale production of commercial hEGF protein.
